# Case report: Prone positioning in the improvement of severe post-operative hypoxia following aortic dissection

**DOI:** 10.3389/fmed.2024.1379128

**Published:** 2024-05-21

**Authors:** Yun Wang, Xuping Cheng, Xuandong Jiang, Lijun Chen

**Affiliations:** ^1^Intensive Care Unit, Affiliated Dongyang Hospital of Wenzhou Medical University, Dongyang, Zhejiang, China; ^2^Cardiac Surgery, Affiliated Dongyang Hospital of Wenzhou Medical University, Dongyang, Zhejiang, China

**Keywords:** aortic dissection, postoperative hypoxia, prone positioning, acute respiratory distress syndrome, pulmonary embolism

## Abstract

Postoperative hypoxemia after aortic dissection surgery presents a considerable clinical challenge, and acute respiratory distress syndrome (ARDS) is a common etiology. Prone positioning treatment has emerged as a potential intervention for improving respiratory function in this context. We report the case of a 27-year-old male who developed severe hypoxemia complicated by pulmonary embolism after aortic dissection surgery. He was diagnosed with postoperative hypoxemia combined with pulmonary embolism following aortic dissection. His respiratory status continued to deteriorate despite receiving standard postoperative care, thereby necessitating an alternative approach. Implementation of prone positioning treatment led to a substantial amelioration in his oxygenation and overall respiratory health, with a consistent hemodynamic state observed throughout the treatment. This technique resulted in significant relief in symptoms and improvement in respiratory parameters, facilitating successful extubation and, ultimately, discharge. This case underlines the possible efficacy of prone positioning therapy in managing severe hypoxia complicated by pulmonary embolism following aortic dissection surgery, warranting more thorough research to explore the potential of this treatment modality.

## Introduction

Postoperative hypoxemia is a common and challenging complication of type A aortic dissection surgery, and its management is crucial for satisfactory patient outcomes (1, 2). It is often associated with factors such as obesity, smoking history, prolonged extracorporeal circulation, intraoperative transfusions, and systemic inflammatory responses (3). These factors may lead to acute respiratory distress syndrome (ARDS), a severe and life-threatening condition characterized by arterial hypoxemia and diffuse pulmonary infiltrates (4). The incidence of ARDS in patients with type A aortic dissection can reach up to 56%, with severe hypoxemia (PAO2/FIO2 < 100) occurring in approximately 36% of patients (1, 5). In an effort to facilitate early identification of high-risk patients, Gajic et al. recently proposed a lung injury prediction score, which includes factors such as shock, sepsis, high-risk surgery, obesity, hypoalbuminemia, acidosis, and diabetes mellitus (6). The pathophysiological mechanisms underlying postoperative hypoxemia primarily involve the effects of extracorporeal circulation, ischemia–reperfusion injury, and transfusion-related acute lung injury (7). During extracorporeal circulation, reduced pulmonary arterial flow and the release of numerous inflammatory mediators increase pulmonary vascular permeability, leading to lung injury (8). Additionally, transient ischemia of the lungs during cardiac surgery followed by reperfusion can generate harmful reactive oxygen species, and substantial blood transfusions can activate neutrophils, causing damage to alveolar epithelial and endothelial cells, resulting in lung injury and subsequent hypoxemia (9, 10).

Prone positioning, wherein the patient is placed face down, can enhance pulmonary function by optimizing ventilation-perfusion matching, reducing the pressure of the heart on the posterior lungs, and potentially lowering the pressure required to achieve alveolar recruitment (11, 12). While extensively studied and applied in ARDS, the utility of prone positioning in post-cardiac surgery settings (especially after surgery for an aortic dissection) is relatively less documented due to concerns about hemodynamic instability and wound infections after sternotomies (13, 14).

Pulmonary embolism is another serious condition that can exacerbate postoperative hypoxemia. However, the occurrence of pulmonary embolism after cardiac surgery is relatively rare and poses a diagnostic challenge, because the symptoms can be masked by the underlying postoperative state. This case report aims to discuss the innovative application of prone positioning as a salvage therapy for severe hypoxemia secondary to ARDS compounded by pulmonary embolism after aortic dissection surgery, a context wherein its use is not yet well established.

## Case description

A 27-year-old male with a history of hypertension and obesity (weight, 140 kg; height, 182 cm) and no family history of venous thromboembolism was admitted to the hospital on May 29, 2022, with a chief complaint of sudden chest and back pain that had lasted for 6 h. Findings from emergency chest computed tomography (CT) were suggestive of an aortic dissection, leading to an immediate ascending aortic replacement under general anesthesia. This procedure was facilitated by extracorporeal circulation, which lasted for 3 h, while the comprehensive surgical procedure lasted for 10 h. Intraoperatively, the initial aortic dissection rupture was found to be located at the root of the ascending aorta above the opening to the right coronary artery, with thrombus formation in the pseudo-lumen of the ascending aorta and dissection formation in the aortic arch on the side of the lesser curvature. Dissection involvement was observed at the roots of the left common carotid artery and left subclavian artery, and the right coronary artery was dilated. During the operation, substantial amounts of blood products were transfused, including 1.5 units of red blood cells and 600 mL of plasma. The patient was transferred to the intensive care unit (ICU) for monitoring and treatment. However, he developed severe intraoperative and postoperative hypoxemia. Mechanical ventilation provided an oxygen concentration pattern of 100% [assist-control/pressure-control (PC), f 20 beats/min; PC, 18 cmH_2_O; and positive end-expiratory pressure (PEEP), 12 cmH_2_O]; however, the oxygenation index gradually decreased (reaching a minimum of 41 on day 3), as did the oxygen saturation (72%), resulting in systemic hypoxia. The blood gas analysis results were as follows: pH, 7.43; pCO_2_, 38.9 mmHg; pO_2_, 76.2; HCO_3_, 25.6; and D-dimer, 2.53 μg/mL. The patient was hemodynamically stable.

The patient’s family refused extracorporeal membrane oxygenation therapy due to financial constraints; however, this put the patient at risk of death.

## Diagnostic assessment

Postoperative hypoxemia combined with pulmonary embolism following aortic dissection.

## Physical examination findings

The patient was admitted to the ICU with the following findings: temperature, 39.3°C; blood pressure, 117/64 mmHg; heart rate, 112 beats/min; respiratory rate, 22 breaths/min; oxygen saturation, 96% (ventilator-assisted, 100%; PC, 12 cmH_2_O; tidal volume, 712 mL; and PEEP, 8 cmH_2_O). Physical examination and endotracheal intubation were performed. The lungs were clear, and the heart rhythm was regular. All other findings were unremarkable.

## Imaging examination findings

Radiological examinations were performed; their findings are listed below.

**Figure 1:**
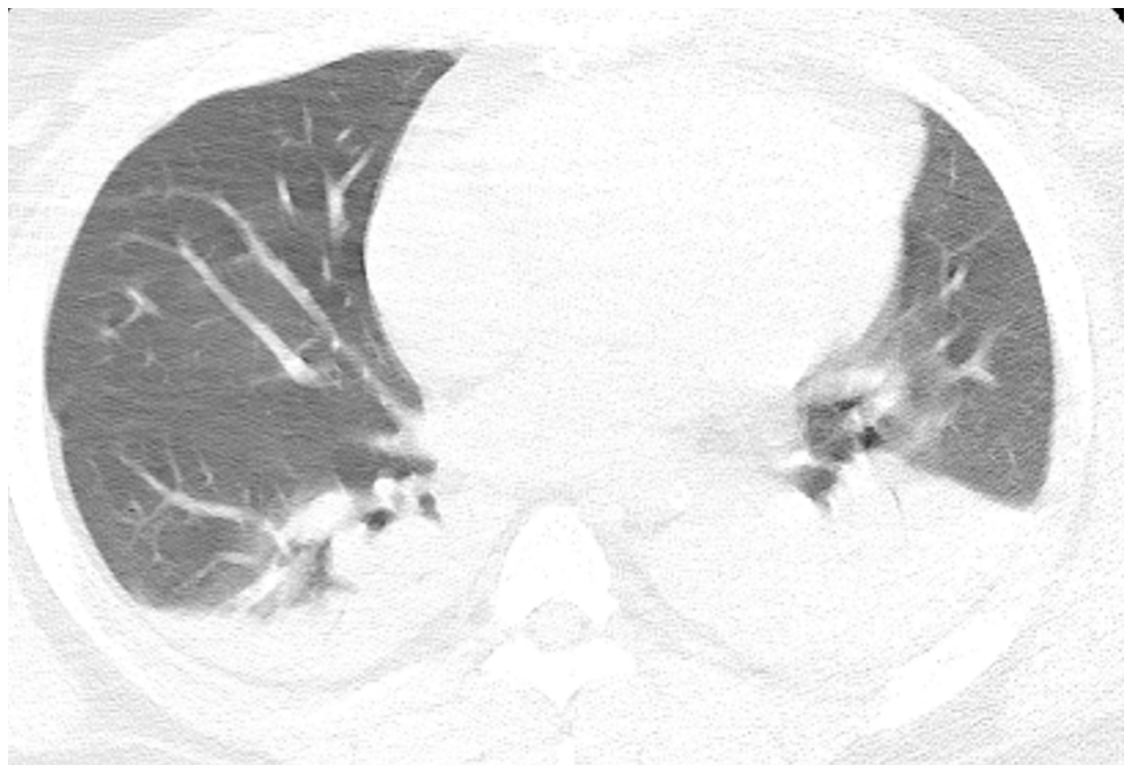
Chest computed tomography reveals small amounts of pleural effusion with atelectasis of the bilateral lower lobes, a small amount of exudate from the upper lobe of the left lung, and a small number of fibrous linear foci in both lungs.

**Figure 2:**
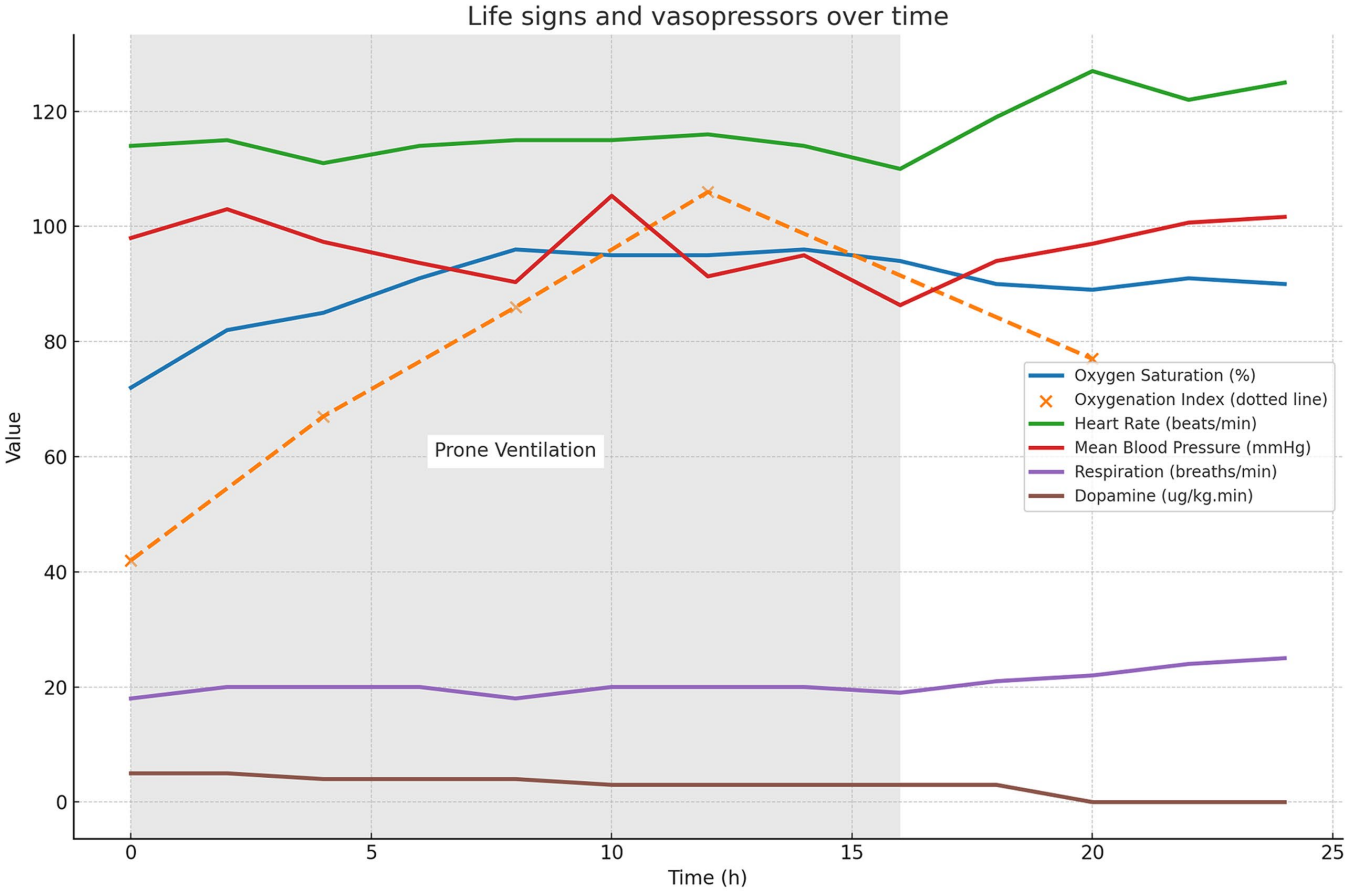
Chest computed tomography angiography (CTA) reveals embolization of both the main trunk of the right pulmonary artery and multiple arterial branches bilaterally.

- Echocardiography: past history of ascending aortic replacement, left ventricular hypertrophy, and mild tricuspid valve regurgitation (ejection fraction: 59%).
- Chest CT: small amounts of pleural effusion with atelectasis of the lower lobes bilaterally, small amounts of exudate from the upper lobe of the left lung, and a small number of fibrous linear foci in both lungs (Figure 1).
- Aortic computed tomography angiography (CTA): embolization of both the main trunk of the right pulmonary artery and multiple arterial branches bilaterally (Figure 2).

Accordingly, the patient was diagnosed with hypoxemia complicated by pulmonary embolism following aortic dissection surgery.

**Figure 3:**
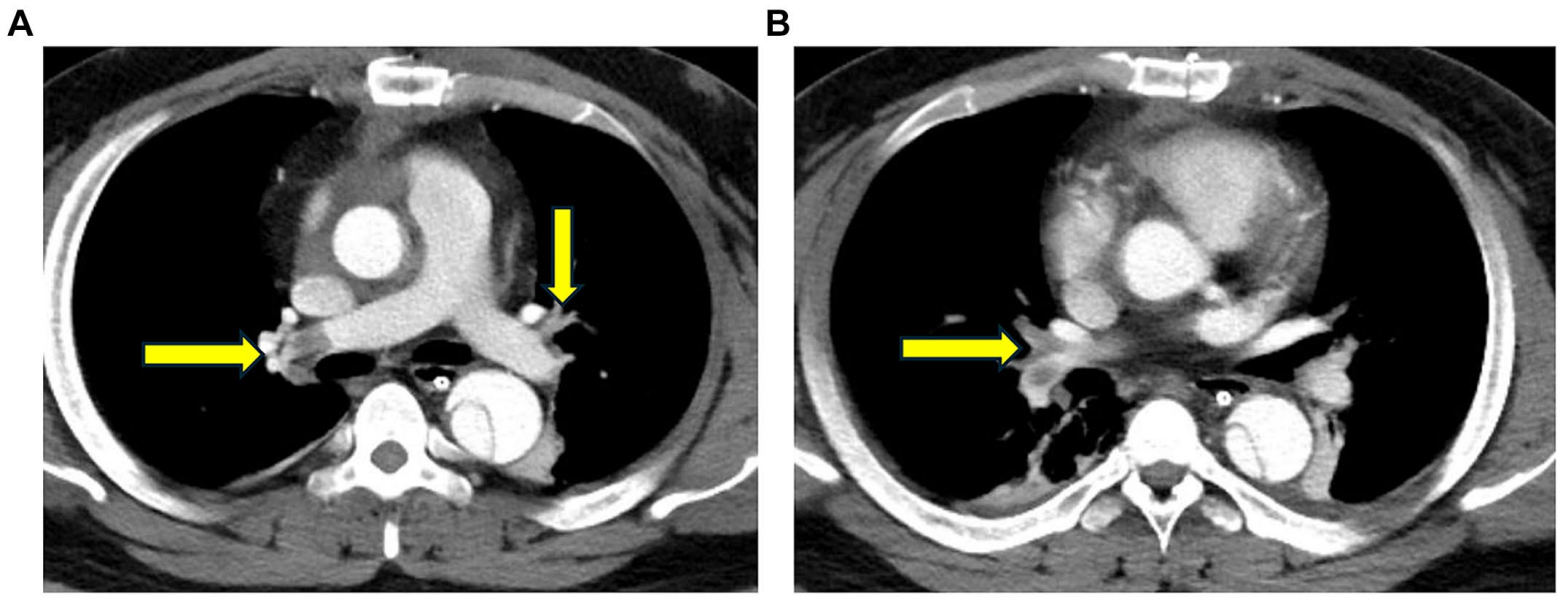
Changes in the oxygen saturation on pulse oximetry, oxygenation index, vital signs, and vasoactive medication usage from before to after prone positioning treatment. The light gray background in the figure indicates the period of ventilation during prone positioning treatment (0–16 h).

With the consent of the patient’s family, prone positioning treatment was initiated on day 3, with the first session lasting for 16 h. To ensure safety during the prone positioning session, we assembled a multidisciplinary team comprising 6–8 members, including respiratory therapists, ICU physicians, nurses, and rehabilitation therapists. Following this treatment, the patient’s oxygen saturation gradually increased to 96%, and the oxygenation index increased to 121. Figure 3 illustrates the changes in oxygen saturation (measured by pulse oximetry), oxygenation index, vital signs, and usage of vasoactive medication from before to after the prone positioning treatment. These oxygenation indicators showed significant improvement, and the patient remained relatively hemodynamically stable. These oxygenation markers exhibited a significant improvement, and the patient maintained a relatively stable hemodynamic condition without the occurrence of any other severe complications. This clinical strategy also included thrombectomy and extubation, in conjunction with heparin and warfarin anticoagulation therapy, initiated on day 9. The patient was discharged on day 35.

## Follow-up outcomes

During the 1-month post-discharge follow-up, the patient’s condition was stable, with cardiopulmonary functions returning to preoperative levels. Furthermore, no definitive pulmonary embolism in the pulmonary artery was observed in the chest computed tomography angiography (CTA).

## Discussion

We present a unique case of severe hypoxemia due to ARDS, compounded by pulmonary embolism following aortic dissection surgery, which was successfully managed with prone positioning treatment. While not the definitive treatment for these complications, prone positioning was chosen for its physiological benefits. This intervention is well established in ARDS management but is less frequently considered after cardiac surgery due to the risk of hemodynamic instability and wound complications. Such cases are extremely rare, and only one similar case has been reported by Cheong et al. This previous case involved a 57-year-old male who developed pulmonary embolism combined with severe hypoxemia after gastric bypass surgery, which improved with mechanical ventilation in the prone position (15). In addition, echocardiography confirmed that prone positioning treatment improved the patient’s right ventricular dysfunction. The present case demonstrates the feasibility and potential benefits of prone positioning treatment even in complex postoperative settings.

The imaging findings in the present case provide an important overview of the patient’s condition and complications following aortic dissection surgery. CT of the patient’s lungs revealed small amounts of pleural effusion with atelectasis of the lower lobes bilaterally (Figure 1). Furthermore, a small amount of exudate from the upper lobe of the left lung was observed, in addition to a small number of fibrous linear foci in both lungs. These finding are typical of ARDS, which partially explains the development of postoperative hypoxemia. However, ARDS alone does not fully account for the severe hypoxia observed in our patient, as indicated by an oxygenation index of 41. Computed tomography angiography (CTA) was performed, suspecting pulmonary embolism as the primary cause of the pronounced hypoxemia. Nevertheless, due to the absence of a patent foramen ovale assessment at that time, a definitive correlation between the hypoxemia and embolic events could not be confirmed. Establishing this diagnosis highlighted the need for additional investigations and targeted therapeutic measures for pulmonary embolism in response to severe postoperative hypoxemia. Concomitant pulmonary embolism, which exacerbates hypoxemia, is associated with an altered ventilation/perfusion ratio (V/Q) due to mechanical and functional obstruction of the vascular bed and with inflammatory responses leading to surfactant dysfunction, pulmonary atrophy, and intrapulmonary shunt (16). The absence of right heart catheterization assessments before and after prone positioning means we can only speculate that the treatment’s mechanism for ameliorating hypoxemia primarily involves: prone positioning reduces pulmonary vascular resistance and improves oxygenation, thereby alleviating hypoxic vasoconstriction in the pulmonary vascular bed (17).

Hypoxemia following aortic dissection surgery, which may include concomitant pulmonary embolism, is often missed. The D-dimer level may not be a good indicator of pulmonary embolism because patients with aortic dissection can inherently have elevated D-dimer levels. Postoperative hypoxemia in cases of aortic dissection causes pulmonary hypertension and right heart dysfunction, leading to nonspecific ultrasound indications. In the present case, the diagnosis was confirmed by a pulmonary vascular CTA examination. Severe pulmonary embolism can be treated with extracorporeal membrane oxygenation, and surgical thrombolysis can improve morbidity and mortality rates, making surgical thrombolysis an option for patients meeting the appropriate criteria (18).

The safety of prone positioning treatment after cardiac procedures is critically reviewed due to concerns regarding its influence on hemodynamics. Prone positioning increases intra-abdominal pressure and venous return, potentially impacting cardiac filling and output. This may intensify the load on the right heart, especially in patients with unstable pulmonary embolism and right ventricular dysfunction (19). Nevertheless, prone positioning could enhance pulmonary absorption, potentially alleviating hypoxemia, hypercapnia, driving pressure, and plateau pressure, which might improve right ventricular function and overall hemodynamics. However, the effects of prone positioning on hemodynamics and specifically on right ventricular function have not been conclusively determined (20). Additionally, prone positioning may lead to complications such as device displacement, loss of venous access, accidental extubation, endotracheal tube displacement and obstruction, brachial plexus injury, and pressure ulcers (21). Despite these risks, the therapeutic outcomes from prone positioning in patients with ARDS have been more favorable. Gu et al. have substantiated the safety of this practice following aortic dissection surgery (22). The patient in the present case exhibited stable hemodynamics before and after prone positioning treatment, indicating its potential benefits. Successful application of this technique requires an experienced medical team attentively monitoring the patient’s vital signs and oxygenation levels.

## Conclusion

This case highlights the potential of prone positioning as a management strategy for severe postoperative hypoxemia following aortic dissection surgery. It draws attention to the plausible co-presence of pulmonary embolism, underlining that usual clinical indicators might not suffice for diagnosis. Although not a direct treatment for pulmonary embolism, prone positioning, as this case exhibits, can significantly improve respiratory parameters. It demands careful execution by an experienced medical team but can offer considerable benefits post-cardiac surgery.

## Data availability statement

The original contributions presented in the study are included in the article/supplementary material, further inquiries can be directed to the corresponding author.

## Ethics statement

The studies involving humans were approved by the Ethics Committee of the Dongyang People’s Hospital (Dong Ren Yi 2023-YX-286 and date of approval: 09/08/2023). The studies were conducted in accordance with the local legislation and institutional requirements. The participants provided their written informed consent to participate in this study. Written informed consent was obtained from the individual(s) for the publication of any potentially identifiable images or data included in this article.

## Author contributions

YW: Investigation, Resources, Writing – original draft. XC: Conceptualization, Writing – review & editing. XJ: Visualization, Writing – review & editing. LC: Resources, Writing – review & editing.
